# Trial staff and community member perceptions of barriers and solutions to improving racial and ethnic diversity in clinical trial participation; a mixed method study

**DOI:** 10.1016/j.conctc.2024.101262

**Published:** 2024-01-17

**Authors:** Maureen Shields, Anne Rivelli, Yamilé Molina, Osondi Ozoani-Lohrer, Cheryl Lefaiver, Marybeth Ingle, Veronica Fitzpatrick

**Affiliations:** a Advocate Aurora Research Institute, Milwaukee, WI, USA; b Advocate Health, Milwaukee, WI, USA; c University of Illinois, Chicago, IL, USA

**Keywords:** Clinical trials participation, Diversity in clinical trials, Barriers to participation, Solutions for participation, Mixed method

## Abstract

**Background:**

The lack of racial and ethnic diversity in clinical trials leads to skewed findings, limited generalizability, inequitable health outcomes for people of color, and insufficient access to innovative therapies. Our objective was to compare perceptions of barriers to participation in trials for people of color and trial staff to provide tangible solutions for improving diversity among study participants.

**Methods:**

This mixed method study utilized semi-structured interviews and surveys to evaluate barriers to participation and solutions to improve racial and ethnic diversity in clinical trials among healthcare system trial staff and community members from the same region. Through thematic analysis via coded transcripts and quantitative analysis via survey data, social support theory constructs were identified to evaluate where perceptions of barriers and solutions overlap and where they diverge.

**Results:**

A total of 55 trial staff and 75 community members participated in the study. Trial staff identified logistics and patients’ unwillingness to receive additional treatments as perceived barriers to participation, while community members stated lack of information and lack of trust in their care team. Both groups identified hesitance toward research as a prominent barrier. Solutions related to informational support demonstrated the most overlap between groups, while instrumental support showed the most discordance.

**Conclusion:**

Solutions for improving racial and ethnic diversity in clinical trial participation are multi-faceted and have various levels of impact. Overlap and discordance of opinions regarding solutions should be further evaluated, and implementation of solutions should be carefully considered.

## Highlights

• Barriers and solutions among trial staff and community members were analyzed.
• Informational and emotional constructs overlapped between the two groups.
• Instrumental and appraisal constructs diverged between the two groups.
• Access to translation services emerged as a prominent health system barrier.
• Increasing awareness and education of trials may lead to more equitable enrollment.

## 1 Introduction

Despite recent efforts made to diversify trial participation [[1], [2], [3]], clinical trials (“trials”) still lack racial and ethnic diversity [[4], [5], [6], [7]]. Overall, trial participants tend to be non-Hispanic White (NHW) individuals, resulting in underrepresentation of non-White individuals [[8], [9], [10]]. Homogenous trial participants lead to skewed findings, limited generalizability [4,5,11], and disparities in health conditions and outcomes, access to medical care and innovative therapist, and overall scientific knowledge among people of color as compared to their NHW counterparts [6,7,11]. Trials as recent as those studying the COVID-19 pandemic are no exception to the lack of racial and ethnic diversity in participation, despite the stark differences in health outcomes that emerged between people of color and NHW individuals in both COVID-19 related morbidity and mortality [[12], [13], [14]]. To make meaningful changes, it is critical to comprehensively understand barriers to non-White patient participation.

Despite historical beliefs that people of color lack interest in participating in clinical trials [4], researchers have found that this issue is much more complex. Studies show Non-Hispanic Black (NHB) and Hispanic individuals in the US are just as likely as White individuals to confirm their willingness to participate in a trial, suggesting more tangible barriers to participation [4,5,15]. Numerous recent studies have identified barriers to participation among patients approached for trials, including hesitance toward research, transportation issues, lack of trial awareness, fear of adverse events, insurance issues, influence from family/friends, and more [11,[16], [17], [18], [19], [20], [21]]. Medical comorbidities are another baseline factor that create disparity in participation through access, reflected by NHB men being excluded from trials due to significantly more comorbidities [6]. Research has suggested that the design of future trials be more conservative with exclusion criteria to enroll more diverse patients [6].

Research has also identified patient-, provider-, and administrative-level barriers to enrolling people of color. At the patient level, health literacy, poverty, patient burden, negative attitudes toward research, and perceived gravity of the medical condition are deterrents to enrollment [[22], [23], [24]]. At the physician level, lack of time, knowledge of trial details, and incentives/rewards have been identified [23,24]. Finally, administrative barriers to enrollment include limited availability of translation services and lack of appropriate trials for community-based settings [22,23].

Interestingly, barriers faced specifically by enrolling clinical trial staff are rarely addressed in the literature, as most barriers reflect on participants’ observations of research [22]. In the limited research conducted among trial staff, patient education, community outreach, additional support personnel, and site education emerge as potential solutions to improving enrollment of people of color [22,23]. Trial staff are crucial in developing, organizing, and implementing clinical trials, and should be given more opportunities to share their perspectives on enhancing clinical trial processes [22,25]. Their relationships with patients paired with their awareness of nuances of the healthcare system allow them to assess barriers and solutions from unique angles [23]. Additional research is needed to better understand how investigators, coordinators, and other clinical trial staff can improve strategies for enrolling people of color, and how those solutions overlap and/or deviate from community-centered solutions.

The purpose of this study is to: 1) identify barriers and solutions to equitable participation in trials from the perspective of self-identified people of color and/or Hispanic persons as well as clinical trial staff and 2) compare perceptions of barriers and solutions between the two participant groups. This study used a mixed method approach to capture data from both healthcare system trial staff and community members from the healthcare system catchment area. The responses were aggregated separately (trial staff and community members) then compared to each other as a way to determine how much overlap and difference there is in perceptions of the problem (barriers) and solutions.

## 2 Materials and methods

This mixed methods prospective study utilized data collected via semi-structured interviews and surveys between 3/14/2022 and 12/14/2022 in a large midwestern non-profit healthcare system. Data was prospectively collected by the study team from two stakeholder groups during this timeframe: Staff who led, coordinated, or were otherwise involved in a clinical trial in the past 5 years (“trial staff”) and community members residing in the healthcare system's catchment area (“community members”).

An exploratory sequential mixed method strategy was used to employ qualitative and quantitative data collection and analysis methods to determine both stakeholder groups’ perceptions and experiences related to clinical trial participation. The exploratory design begins with qualitative data collection and analysis followed by quantitative data collection and analysis [[26], [27], [28]]. In this study, trial staff interviews were conducted in the initial stage of the project. Their interview responses were analyzed and used to populate multiple choice answer options regarding barriers and solutions in the survey for subsequent trial staff and community member participants. Following initial trial staff interviews, trial staff surveys were then conducted followed by interviews and administration of surveys among community members concurrently. The use of semi-structured interviews allowed the study team to capture rich, in-depth information regarding trial participation, while the use of surveys allowed the study team to efficiently capture quantitative data from those who may not have had the capacity and/or interest to complete an interview.

Grounded theory was used to guide construct and analysis of themes found in the interview data. Where possible, qualitative interview data was coded to match answer options of the survey so the interview data could be integrated into the survey data set, thereby creating one data set with exclusively quantitative variables to be analyzed to address our research questions. Additionally, interviews were separately analyzed to capture the robustness and integrity of interview responses. Informed consent was obtained electronically from all trial staff and community members prior to their participation through the Research Electronic Data Capture (REDCap) e-Consent feature [29,30]. This study was approved by the healthcare system institutional review board: #22.017E.

### 2.1 Identification/recruitment

#### 2.1.1 Trial staff

Trial staff were recruited through an email request to clinical trial department leaders to participate in a 30-min virtual interview. To strengthen recruitment, leaders were asked to communicate the opportunity to their staff. Those who indicated interest were contacted by the study team to schedule the interview. Once a date and time for the interview was confirmed, an electronic calendar invite was sent with a hyperlink to the REDCap e-Consent form for trial staff participants to review and sign. After interviews with trial staff concluded, a REDCap survey was sent to remaining eligible clinical trial staff. Eligibility criteria for trial staff participants consisted of being currently employed within the healthcare system at the time of recruitment and have led, coordinated, or been involved in a trial in any capacity in the past 5 years (self-identified).Trial staff participants were not compensated for their participation.

#### 2.1.2 Community members

Community members were recruited via flyers containing a QR code at previously established health events (e.g., health fair) run by the healthcare system's Community Health Department. To improve recruitment, approached and enrolled community members were encouraged to give flyers to friends and family. The QR code led to a REDCap landing page where potential participants were screened for eligibility and offered either an interview or online survey. If the community member opted for an interview, they were prompted to enter their name, phone number, and email address so a study team member could contact them to schedule the interview. If the community member opted for the survey, REDCap directed them to a new page to review and sign the e-Consent form. Once consented, the participant was led to the survey, also in REDCap. Community member participants needed to be at least 18 years old, willing to share their perceptions of research, able to read and write in English or Spanish, and have an active email address. Identifying as a person of color was initially a requirement, but the study team broadened inclusion criteria to account for those who may have insight due to cultural differences unrelated to race or ethnicity. Community member participants were compensated $20 for 20 min survey or $50 for 30-min interview.

### 2.2 Data sources

Both interviews and surveys were created by the study team members and covered the same topic areas, described below in Table 1. All interviews were conducted via an online video conference platform by at least two members of the study team. Survey data was collected via REDCap, and a complete list of questions can be found in Appendix A.

**Table 1:** Interview and survey domains.

Trial staff	
Domain	Purpose
Your Role in Trials	Aimed to gather information about trial staff and their experience working in trials research.
Trial Processes and Recruitment	Evaluated screening and recruitment logistics, common inclusion and exclusion criteria, and perceptions of demographic representation of study samples.
Barriers	Examined how trial staff view the disparity between who is burdened with diseases versus who accesses and enrolls in trials. The study team asked participants to identify barriers from the perspective of the patient as well as from their own experience enrolling participants.
COVID-19	Assessed how many trial staff were involved in COVID-19 trials. The study team also asked about study design, recruitment, and/or staffing changes that trial staff experienced during this time.
Solutions	Provided tangible solutions to encourage underrepresented people to enroll in trials. Interview participants were asked to offer ideas for both clinic- and system-level solutions.
Demographics	Demographics of trial staff were not relevant to the study, so only race/ethnicity was collected and only from survey participants. To prevent trial staff from feeling uncomfortable, race/ethnicity was not asked of interview participants.

Community Members	
Domain	Purpose and Activity (if Applicable)
Perceptions of Health Care	Assessed general experiences with and feelings toward health care. Interview participants completed a virtual projective activity in which they were asked to select the two emojis that best describe their experience with health care. Survey participants were asked for this information in the form of emotions listed in a multiple-choice question instead of a virtual activity.
Trials Participation	Assessed the participant's familiarity with trials research and provided an opportunity to indicate whether they or anyone they knew had participated in a trial and what that experience was like.
COVID-19 Trials Participation	Assessed potential increased knowledge of trial research due to COVID-19 and provided an opportunity for participants to indicate whether they would participate in a COVID-19 trial.
Barriers	Assessed what barriers a person might experience when participating/trying to participate in a trial based on one's own experience or theoretical barriers. Multiple-choice options were available for survey respondents, as was an open text box for barriers not already listed.
Solutions	Focused on implementable solutions for reducing barriers and encouraging trial participation. Interview participants completed a virtual card sorting activity in which they were asked to rank solutions already identified by trial staff participants. Solution options were available for survey respondents as multiple-choice options and an open text box for solutions not already listed.
Demographics	Collected during interviews and surveys, including sex, race/ethnicity, age, insurance type, and zip code.

### 2.3 Data analysis

#### 2.3.1 Qualitative

Thematic analysis was conducted to evaluate the central purpose of this study: to identify barriers and solutions to participating in trials. Interviews were transcribed by a professional transcription service and read thoroughly by study team members. Response codes were used to organize qualitative data and identify themes, and in the context of this study reflected the barriers and solutions presented to trial staff and community members during interviews and surveys. Response code definitions were created a priori based on literature review and trial staff interview findings. A complete list of response codes can be found in Appendix B. The same barriers were presented to team and community members and can therefore be easily compared. Solutions presented to the two groups, however, were not always the same. The study team decided together that certain solutions identified by trial staff were irrelevant to community members and their experiences with trials and, thus, were not presented (e.g., change physician compensation model). The study team selected a group of relevant solutions, based on a literature review, from which community members could choose. Deductive coding was used to apply response codes to excerpts related to trial participation barriers as well as potential solutions for making trial enrollment more equitable and successful. Simultaneous coding was used at times, as certain excerpts may have included more than one theme and were therefore coded with multiple codes. Coding was done independently by two members of the study team to ensure data credibility.

Lastly, due to the numerous and wide range of response codes, the study team conducted an activity to group response codes as Social Support Theory constructs (constructs) for brevity and digestibility of results. Social support theory is a behavioral theory that concerns itself with social relationships and is often applied to situations where it is important to figure out what kind of support people need to act (in this case, participate in a trial) [31]. The theory has four major constructs (not exhaustive), all related to different types of support. Response codes were grouped based on the following definitions.

• Informational: Excerpts that report or imply the need for advice, suggestions, and/or information
• Instrumental: Excerpts that report or imply the need for a tangible aid and/or service
• Appraisal: Excerpts that report or imply information that is useful for self-evaluation
• Emotional: Excerpts that report or imply expressions of empathy, love, trust, and care

This process was performed as follows: each of the three primary research study team members (authors one, two and six) independently mapped each response code to the two constructs they felt were the best fit, one identified as primary and the other as back-up. Then, if at least two out of the three study team members matched the response code to the same primary or back-up construct, that response code was grouped under that construct theme. If at least two study team members mapped the response code to two different constructs, the three study team members verbally discussed the best construct and came to a joint decision on which construct theme with which to group that response code.

#### 2.3.2 Quantitative

Univariate analyses were conducted to examine the distribution of demographic variables among the sample. Descriptive statistics are reported as counts (%) for categorical variables and mean (SD) for continuous variables. Heat maps were created using the Color Scales feature in Excel to highlight proportions of perceived barriers to participation and proposed solutions to increasing diversity in trial participation as identified by community members and trial staff. Barriers and solutions with the highest rates of endorsement are colored red, intermediate-high rates of endorsement are colored orange, low-intermediate rates of endorsement are colored yellow, and low rates of endorsement are colored green. Once social support theory construct groupings were finalized, counts (%) of trial staff and community member participants who identified the response codes and, thus, the derived constructs were evaluated. Participants within each group could have identified multiple response codes, thus columns do not equal 100 %. Bivariate analyses were conducted to examine differences in social support theory constructs between trial staff participants and community member participants. Reported measures of association include odds ratios (OR) with associated 95 % confidence intervals (CI). Corresponding p-values were generated from Chi square tests to assess statistically significant differences in suggesting a barrier construct or a solution construct. Excel was used for data management by the study team and statistical analysis was performed using SAS statistical software (Version 9.4; SAS Institute, Cary, NC). Alpha of p < 0.05 was considered statistically significant in all analyses.

## 3 Results

Among the 55 trial staff participants, 42 (76.36 %) completed surveys and 13 (23.63 %) completed interviews (Table 2). Overall, most participants were Clinical Research Coordinators (50.91%) who worked on more than one service line (36.36 %), at an urban site (50.91 %) in Illinois (50.91 %) and identified as NHW (56.36 %). The mean number of years worked in trials was 9.92 (±7.55). Among the 75 community member participants, 55 (73.33 %) completed surveys and 20 (26.67 %) completed interviews (Table 2). Overall, most participants were female (74.67 %), NHB (44.00 %), had private insurance through their job (46.67 %), were a mean age of 49, and resided in Chicago, Illinois (48.00 %).

**Table 2:** Characteristics of participants.

Variables^a^	Trial staff	Community Members
	N = 55	N = 75
**Data Type**		
Survey	42 (76.36 %)	55 (73.33 %)
Interview	13 (23.64 %)	20 (26.67 %)
**Race & Ethnicity**		
NHW	31 (56.36 %)	3 (4.00 %)
NHB	2 (3.64 %)	33 (44.00 %)
Hispanic or Latino	0	27 (36.00 %)
Asian	0	10 (13.33 %)
More than one race	2 (3.64 %)	0
Unknown^b^	20 (36.36%)	2 (2.67%)
**Geographic Location**		
IL	28 (50.91 %)	59 (78.67 %)
Chicago	–	36 (48.00 %)
Non-Chicago (Chicago suburb)	–	23 (30.67 %)
WI (Milwaukee)	23 (41.82 %)	7 (9.33 %)
Unknown^c^	4 (7.27 %)	9 (12.00%)
**Age [Mean (SD)]; [Median (IQR)]**	–	49.19 (18.03); 49.50 (30)
**Sex**		
Female	–	56 (74.67 %)
Male	–	19 (25.33 %)
**Insurance**		
Medicare/Medicaid	–	27 (36.00 %)
Insurance through my job	–	35 (46.67 %)
Insurance through my partner's job	–	7 (9.33 %)
Insurance through the Marketplace/exchange	–	1 (1.33 %)
Unknown	55 (100.00 %)	5 (6.67 %)
**Role in clinical trials**		
Administrative	6 (10.91 %)	–
Research Assistant or Coordinator	28 (50.91%)	–
PI/Sub-I/Co–I	2 (3.64 %)	–
More than one role	14 (25.45 %)	–
Other	5 (9.09 %)	–
**Service Line within clinical trials (N = 55)**		
Cardiology	9 (16.36 %)	–
Neurology	3 (5.45 %)	–
Oncology	15 (27.27 %)	–
Pediatrics	3 (5.45 %)	–
More than one service line	20 (36.36 %)	–
Other	5 (9.09 %)	–
**Years worked in clinical trials, [Mean (SD)]; [Median (IQR)]**	9.92 (7.55); 8.00 (11.00)	–

a Rows with “-“ indicate items that were not asked of participants in that column, either for lack of relevance or to mitigate potential discomfort.

b Trial staff who were interviewed were not asked race/ethnicity (13/20 missing here).

c Unknown site location among trial staff refers to remote workers.

### 3.1 Perceived barriers to participation

Among trial staff, the three most frequently identified perceived barriers to increasing racial and ethnic diversity in clinical trial participants were: 1) general hesitance toward research, 2) patients not willing to receive additional procedures or treatments, and 3) logistics. Among community members, the three most frequently identified barriers to participating were: 1) lack of information and awareness, 2) lack of trust in care team, and 3) general hesitance toward research. Proportions of all perceived barriers to participation are displayed as a heat map in Table 3.

**Table 3:**
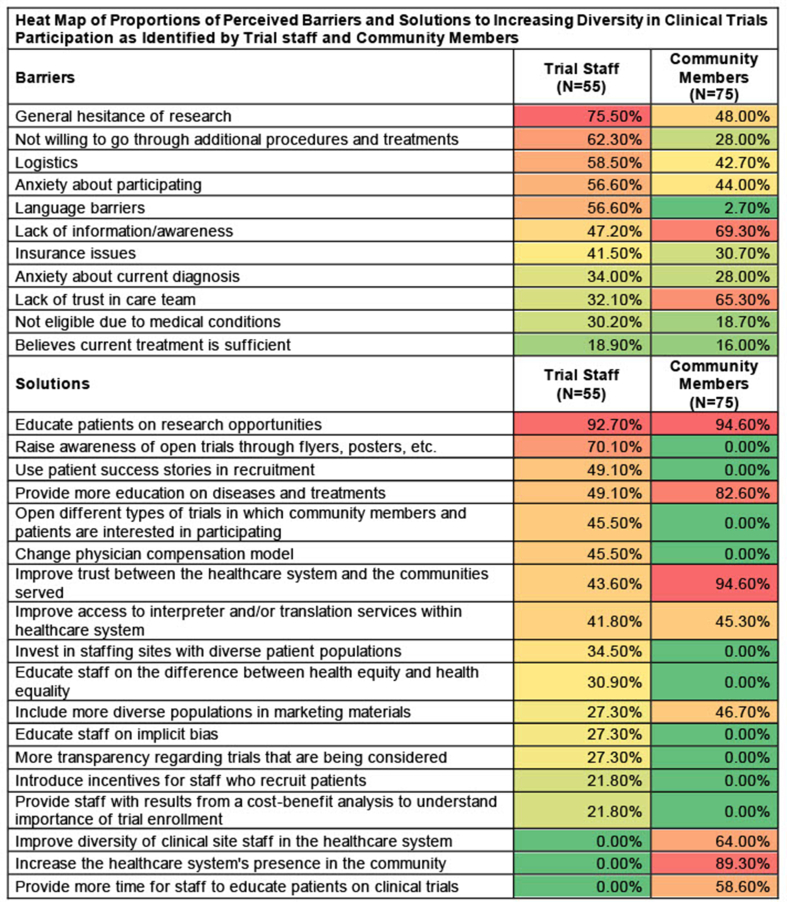
Heat Map of Proportions of Perceived Barriers and Solutions to Increasing Diversity in Clinical Trials Participation as Identified by Trial staff and Community Members.

### 3.2 Perceived solutions to participation

While identifying barriers to participation is crucial, we focus on results related to potential solutions for the remainder of this paper, as we feel it is important to understand where perceived solutions overlap and diverge between trial staff and community members. Solutions most frequently identified by trial staff in our sample were: 1) educating patients on research opportunities, 2) raising awareness of open trials through tangible materials like flyers and posters, and 3) using patient success stories in recruitment as well as 3) educating patients on disease basics and expected outcomes (tied due to the same number of response codes applied). Community member participants identified: 1) increasing awareness of trial opportunities, 2) improving trust between the healthcare system and the communities served, and 3) increasing the healthcare system's presence in the community as solutions thought to improve inclusivity in trial participation. Proportions of all proposed solutions are displayed as a heat map in Table 3. Solutions with only one proportion reflect those identified by only one group.

### 3.3 Social support theory

**Fig. 1:**
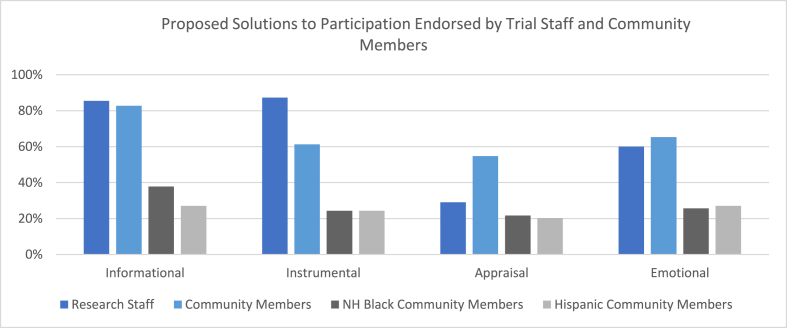
Proposed solutions to participation endorsed by trial staff and community members.

A derivation of the Social Support Theory was used to create a framework around the individual response items. Table 4 presents the individual response codes grouped using the social support theory constructs among trial staff and community member participants. Counts (%) of participants endorsing each construct and relevant sample quotes from participants to provide additional qualitative context to our findings are included in the table as well. Because some solution response codes differed between trial staff and community members, Table 4 outlines separate construct groupings for each participant group. Fig. 1 presents proposed solutions as constructs to illustrate where team and community member solutions overlap and diverge. Given that the barriers and solutions to trial participation may vary based on race/ethnicity, the constructs for community member participants were categorized overall and by race.

**Table 4:** Grouping of Solution Codes to form Social Support Theory Constructs.

Trial Staff		
Initial codes grouped to form construct		N (%) of Trial Staff Endorsing Theme (N = 55)
**Construct 1: Informational Support**		
• Educate patients on research opportunities • Raise awareness of open trials through flyers, posters, etc. • Educate patients on disease basics and expected outcomes • Educate staff on the difference between health equity and health equality • Provide staff with results from a cost-benefit analysis to understand importance of trial enrollment • Educate staff on implicit bias		47 (85.5%)
*I mean, definitely breaking the stigma around it with education is the biggest one. I would probably say approaching patients when they first become patients. And just letting them know we do participate in clinical trials and research here. At some point, if we think it's best for your health and best for your treatment that you might be approached about it. – TS05*		
**Construct 2: Instrumental Support**		
• Improve system-wide access to interpreter and/or translation services • Change physician compensation model • Open different types of trials in which community members and patients are interested in participating • Invest in staffing sites with diverse patient populations • Introduce incentives for staff who recruit patients • Include more diverse populations in AAH marketing materials		48 (87.3%)
*That's the thing at one of the Illinois sites as well. It's one of the few that's positioned in the metro area and they don't get the correct studies it seems. More of the studies are going to another hospital which is predominantly white. Just because they know they can get the numbers. – TS12* *For physicians who spend a lot of their clinic time talking about clinical trials, and ultimately accruing those patients to clinical trials, there should be some incentive for that. But as of today, there is not. – TS06*		
**Construct 3: Appraisal Support**		
• More transparency regarding trials that are being considered		16 (29.1%)
*There has to be a centralized way for trial staff to know what trials are being looked at. Which ones are in the pipeline, and what training/work is left, and all currently active trials, et cetera. Coordinators are the boots on the ground, they're the ones that are going to be enrolling your patients. So it only behooves them to let them know what's going on. When you're down the road with this trial that isn't successful. And you're like, "If you just would've given me a seat at the table, I could have told you that." – TS04*		
**Construct 4: Emotional Support**		
• Use patient success stories in recruitment • Find ways to foster trust between the system and the communities served		33 (60.0%)
*There's so many different things that we can actually get involved in our community and help them live well. There are so many things where the healthcare system can actually gain trust within their community, as being part of the community, rather than we're only here for you when something goes wrong. – TS06*		
**Community Members**		
**Initial codes grouped to form construct**	**N (%) of Community Members Endorsing Theme (N** = **75)**	
**Construct 1: Informational Support**		
• Provide more education on diseases and treatments • Increase awareness of trial opportunities	62 (82.7%)	
*Many times, these things present themselves only after you have some type of sickness or condition. There should just be general discussion early on. – CM15*		
**Construct 2: Instrumental Support**		
• Improve access to interpreter and/or translation services within the healthcare system • Provide more time for staff to educate patients on clinical trials	46 (61.3%)	
*Include more languages because it's not just English or English and Spanish. So, just be more diverse in that area to have people who understand and can conduct the trials in other languages. Just be more inclusive as to who they can ask to participate or have a wider range of people who can participate. – CM31*		
**Construct 3: Appraisal Support**		
• Improve diversity of clinical site staff in the health system • Include more diverse populations in marketing materials	41 (54.7%)	
*You got to have somebody like me to come tell a story. The real personal stories are ones that help other people say, "Okay. Well, she's got that experience." – CM11*		
**Construct 4: Emotional Support**		
• Improve trust between the healthcare system and the communities served	49 (65.3%)	
*People got to speak to them, talk to them. If they go to different things where you get a lot of Black participation every night, then have somebody get up and speak about these studies, about how it's going to be helpful to your children, your grandkids, your future, your generation. It's going to help. You got to help them to trust you. So more exposure is better than anything. I don't think it's been exposed to our community that much. – CM20*

#### 3.3.1 Social support construct: informational support

Among trial staff participants, 47 (85.5 %) endorsed a solution to improving racial and ethnic diversity in clinical trials that mapped to an informational support construct. Informational support included anything related to providing more information about clinical trials, how to locate clinical trials, and education on various related topics such as health equity vs. health equality and implicit bias. 62 of the 75 community members (82.7 %) agreed that informational support would improve the likelihood of participating in a trial. Informational support was the most frequently cited construct needed to improve racial and ethnic diversity in clinical trials among community member participants and was the second-most-frequently identified construct among trial staff (by 1). Informational support construct solutions had the most overlap between trial staff and community members. Additionally, both NHB and Hispanic community members cited informational support as most needed to participate in a clinical trial. In the bivariate analysis, presented in Table 5, the odds for trial staff participants to identify an informational support theory construct are 1.23 times those of community member participants (OR = 1.23, 95 % CI: 0.47–3.21), but was not statistically significant (p = 0.6696).

**Table 5:** Bivariate analysis to examine differences in social support theory constructs between trial staff and community member participants.

Support Theory Constructs: Solutions	Overall Sample (N = 130)	Trial staff (N = 55)	Community Members (N = 75)	OR (95%CI)	P-value
Informational	109 (83.85 %)	47 (85.45 %)	62 (82.67 %)	1.23 (0.47–3.21)	0.6696
Instrumental	94 (72.31 %)	48 (87.27 %)	46 (61.33 %)	4.32 (1.72–10.84)	0.0011
Appraisal	57 (43.85 %)	16 (29.09 %)	41 (54.67 %)	0.34 (0.16–0.71)	0.0037
Emotional	82 (63.08 %)	33 (60.00 %)	49 (65.33 %)	0.80 (0.39–1.63)	0.5336

#### 3.3.2 Social support construct: instrumental support

Among trial staff participants, 48 (87.3 %) endorsed a solution to improving racial and ethnic diversity in clinical trials that mapped to an instrumental support construct in the social support theory. Instrumental support included anything related to logistics, staffing, language and communication, and technological solutions related to clinical trials. 46 of the 75 community members (61.3 %) agreed that instrumental support would improve the likelihood of participating in a trial. Instrumental support showed the most discordance between what the trial staff and community members cited as critical solutions to participating in a clinical trial. The odds for trial staff participants to identify an instrumental support theory construct are 4.32 times those of community member participants (OR = 4.32, 95 % CI: 1.72–10.84), which was statistically significant (p = 0.0011) (Table 5).

#### 3.3.3 Social support construct: appraisal support

Among trial staff participants, 16 (29.1 %) endorsed a solution to improving racial and ethnic diversity in clinical trials that mapped to an appraisal support construct in the social support theory. Appraisal support included improved transparency of trials as well as increased visibility of diversity among staff. 41 of the 75 community members (54.7 %) agreed that appraisal support would improve the likelihood of participating in a trial. While individually, items mapped to appraisal support were cited frequently, when aggregating the individual items under the appraisal support construct, both trial staff and community members found this to be least important overall. This includes perceptions from NHB and Hispanic community members. There is also discordance between groups when it comes to perceptions of appraisal support. Trial staff show decreased odds of identifying an appraisal support theory construct compared to community members (OR = 0.34, 95 % CI: 0.16–0.71, p = 0.0037).

#### 3.3.4 Social support construct: emotional support

Among trial staff participants, 33 (60.0 %) endorsed a solution to improving racial and ethnic diversity in clinical trials that mapped to an emotional support construct. Emotional support included anything related to trust, patient success stories, and system and provider support. 49 of the 75 community members (65.3 %) agreed that emotional support would improve the likelihood of participating in a trial, demonstrating overlap between trial staff and community members. Trial staff show decreased odds of identifying an emotional support theory construct compared to community members (OR = 0.80, 95 % CI: 0.39–1.63), however, this finding is not statistically significant (p = 0.5336).

## 4 Discussion

Improving racial and ethnic diversity in clinical trials will take enormous efforts by the healthcare system, trial staff, and individual practitioners. On a healthcare system level, improving trust between people of color and the system is essential but that will require smaller, more manageable actions such as increasing the presence of the healthcare system in the community and improving diversity among trial staff and practitioners. In line with system-based solutions is the improvement in and accessibility of translation services. While only 41.8 % of trial staff and 45.3 % of community members said improving translation services would improve diversity, almost every study participant who identified as Hispanic cited improvement in translation services as essential. While improving diversity among all minoritized groups is necessary, translation services serve as a particularly prominent health system barrier as the need for a translator will invariably increase time with the study recruiter (physician, other clinician, clinical research coordinator) and thus has cascading effects to the individual provider patient volume and workflow. Despite this, accommodations will need to be made if non-English speakers of any race or ethnicity are going to enroll in a clinical trial.

While trust is important, both trial staff and community members cited more information as being critical to participation. Specifically, more information related to what clinical trials are and which are open within the healthcare system, as well as more education on diseases and research opportunities. This is aligned with current research that suggests people of color would participate in a trial if they knew more about them, irrespective of the overall level of trust with healthcare systems [4,5,15]. It was also noted many times that it would be beneficial for information to be provided early in one's healthcare journey, not just when someone is sick and/or eligible for a trial. Further, community members remarked that making information available in their communities and including people of color on recruitment materials and/or informational handouts/websites would help improve the likelihood of participation. This alone may not improve racial and ethnic diversity, but a healthcare system should be able to improve marketing materials and a website more easily than some of the other suggested solutions.

Clinicians and coordinators are uniquely situated to improve racial and ethnic diversity through appraisal and emotional support. Community members cited general hesitance of research, anxiety about participating in something unknown and/or experimental, anxiety about current diagnosis, and an unwillingness to go through additional tests as barriers to participation, all of which can be improved considerate of the relationship between the provider and the patient. As stated earlier, more information will help, but that will not entirely fulfill the desire for more provider support and a better interpersonal relationship with healthcare providers and trial staff, all of which are sure to improve trust.

Opinions regarding informational and emotional constructs demonstrated overlap between trial staff and community members, indicating solutions mapped under these constructs are important to both groups. Health systems should consider prioritizing these types of solutions, as the alignment between staff and patients may lead to more efficient and equitable trial enrollment. The discordance between trial staff and community members regarding instrumental and appraisal constructs should be further evaluated. Among trial staff, instrumental construct solutions such as logistics, staffing, and technology are priorities, as solutions to these barriers may lead to less burdensome enrollment processes. Community members, however, are less concerned with these issues and more focused on receiving information regarding clinical trial opportunities. Appraisal construct solutions such as improved transparency of trials and increased visibility of diversity among staff were more important to community members, as once again the theme of more information emerges as being crucial for participation. Additionally, many community members mentioned feeling better about the process when they saw staff that looked like them. While trial staff acknowledged these solutions as important, they were not as crucial to their own successful enrollment processes. Additional data collection with a focus on these constructs may clarify why these solutions are more important to one group compared to the other. Understanding the discordance is crucial to move forward in creating equitable enrollment strategies and opportunities.

Potential evaluation methods for researchers may include looking quantitatively at clinical trial enrollment numbers to better understand representativeness in trial participation [32]. Analyzing demographic variables such as race/ethnicity, age categories, and insurance type may highlight associations between who is sufficiently represented in certain trials and where gaps may exist. Additionally, researchers may consider evaluating individual clinical trial departments and their specific enrollment processes. The policies and procedures for enrollment in a cardiology trial may be very different from those in a neurology trial, for example. These procedural differences may lead to variations in barriers, which in turn would require a focus on department-specific solutions.

### 4.1 Strengths and limitations

This study contributes valuable data on tangible ways to improve racial and ethnic diversity in clinical trials from both a community member and trial staff perspective. The mixed method design allowed the study team to answer these important research questions with sufficient depth. Community member data were collected by people who may or may not have been patients of the healthcare system, making it more generalizable to the greater community surrounding our healthcare system. Trial staff data are from one community-based healthcare system located in the Midwest, which may not reflect academically affiliated- and/or other healthcare systems with different clinical trials infrastructure. An additional limitation includes the risk that some excerpts may have been miscoded, as the study team had to presume what participants implied through their interview transcripts.

## 5 Conclusion

All the proposed solutions to improving racial and ethnic diversity in clinical trial participation are intertwined and multi-faceted, however, there are small steps that are more easily implemented that may work to achieve the overall goal: improving trust between people of color and the healthcare system. Small implementations such as diversifying recruitment material and webpages related to research are small, tangible actions that may increase participation. Increasing the awareness of available clinical trials and knowledge of what clinical trials entail may lead to increased racial and ethnic diversity in trial enrollment, as might improving the interpersonal relationship between providers and patients. Lastly, implementing effective and efficient translation services for potential participants who prefer a language other than English will be essential.

## Funding

A Study to Explore Disparities in Clinical Trial Participation was funded by the Advocate Charitable Foundation under the COVID-19 Relief Funds Mechanism.

## CRediT authorship contribution statement

**Maureen Shields:** Conceptualization, Data curation, Formal analysis, Visualization, Writing – original draft. **Anne Rivelli:** Validation, Writing – review & editing. **Yamilé Molina:** Writing – review & editing. **Osondi Ozoani-Lohrer:** Data curation, Formal analysis, Resources, Validation. **Cheryl Lefaiver:** Conceptualization, Writing – original draft. **Marybeth Ingle:** Writing – review & editing. **Veronica Fitzpatrick:** Data curation, Funding acquisition, Methodology, Project administration, Writing – original draft.

## Declaration of competing interest

The authors declare that they have no known competing financial interests or personal relationships that could have appeared to influence the work reported in this paper.
