# Supplementary material for: Trial staff and community member perceptions of barriers and solutions to improving racial and ethnic diversity in clinical trial participation; a mixed method study
Source: Contemp Clin Trials Commun. 2024 Jan 17;38:101262. doi: 10.1016/j.conctc.2024.101262 (PMC10847850; doi:10.1016/j.conctc.2024.101262)
Supplement: Multimedia component 1 [file mmc1.docx]

**Appendix A.1: Survey Questions for Trial staff**

**Section 1: Your Role in Clinical Trials (these questions only pertain to your employment at AAH)**

What role(s) have you had while working on clinical trials? Check all that apply.

What service line(s) have you worked for?

How many years of experience do you have working directly in clinical trials?

At which AAH site do you primarily work?

What tasks have you performed while involved in a clinical trial(s)? Check all that apply.

**Section 2: Clinical Trials Processes**

What factors typically determine a study’s inclusion criteria? Check all that apply.

What factors typically determine a study’s exclusion criteria? Check all that apply.

Please select the option that best describes how well you agree with the following statement: The patients that enroll in our clinical trials represent our patient population at the system level.

Please select the option that best describes how well you agree with the following statement: The patients that enroll in our clinical trials represent our patient population at the site/clinic level.

Please select the option that best describes how well you agree with the following statement: The people you enroll in a clinical trial represent the demographics of the condition in the same proportion as the population. For example, even though heart conditions disproportionately impact Black individuals, they account for only 2.5% of clinical trial participants. They also have a 28% higher cancer-specific mortality compared with whites, but only account for 4% of cancer-specific trials. In these instances, the people enrolled do not represent the demographics of the condition.

Please select the option that best describes how well you agree with the following statement: Race and/or ethnicity is an important factor in determining inclusion criteria for a study.

**Section 3: Recruitment**

What strategies have you used to recruit participants for clinical trials? Check all that apply.

Do you ever incorporate multiple strategies when recruiting participants (i.e., community outreach, phone calls, and targeting specific people in inpatient departments for one study)?

Feel free to elaborate on how, in your experience, multiple recruitment strategies have been incorporated

How important is it to your study team to discuss recruitment strategies?

How important is it to you to discuss participant demographics throughout the duration of recruitment?

How often do you inform a patient of a clinical trial opportunity before they have been screened?

How often do you inform a patient that they have screened ineligible for a clinical trial?

How often are monetary and/or material incentives used to promote clinical trials?

**Section 4: Barriers**

What are some barriers you think people experience when it comes to enrolling in clinical trials? Think about this from the perspective of a patient. Check all that apply.

Feel free to provide additional information regarding these barriers here

Do you think all demographic groups experience the same barriers to participation in clinical trials?

Please provide additional information regarding demographic groups experiencing barriers here:

What are common reasons for attrition? Attrition refers to the loss of participants during a study. Check all that you have witnessed and/or heard about.

Please rank the attrition reasons you checked in order of how frequently you’ve witnessed and/or heard about them with your own patient population. [options contingent on previous answer]

What are some barriers you have witnessed and/or heard about when recruiting patients for clinical trials? Think about your own experience enrolling participants. Check all that apply.

Feel free to provide additional information regarding these barriers here: [text box]

**Section 5: COVID-19**

To what extent did study design processes (i.e. protocol development, inclusion/exclusion criteria, etc.) change during COVID-19?

Feel free to provide additional information regarding changes to study design processes during COVID-19 here:

To what extent did recruitment processes change during COVID-19?

Feel free to provide additional information regarding changes to recruitment processes during COVID-19 here:

To what extent did clinical trials staffing needs change during COVID-19?

Feel free to provide additional information regarding staffing needs during COVID-19 here:

What strategies do you use to recruit participants for COVID-19 trials? Check all that apply.

**Section 6: Solutions**

Broadly, what are some things you think we can do to encourage people of color to enroll in clinical trial?

More specifically, what are some things *at your site* we can do to encourage people of color to enroll in clinical trial?

**Section 7: Demographics**

Are you of Hispanic, Latino, or Spanish origin?
What race do you identify as?

**Appendix A.2: Survey Questions for Community Members**

**Section 1: Perceptions of Health Care**

People may have both good and bad feelings about receiving care from doctors, nurses, and other health professionals. Think about experiences you or your loved ones have had, and please check the boxes of all the feelings you associate with receiving care from doctors, nurses, and other health professionals.

Feel free to elaborate on these feelings:

Have you, personally, experienced something significant with your health where you were required to seek care at a clinic or hospital?

If yes, currently or yes, but not recently:

Did you undergo treatment when you experienced something significant with your health?

Were you prescribed any medication?

How frequently did you go to the doctor during that time?

Overall, how was your experience with healthcare during that time? (This could mean your experience with your doctor, the clinic or hospital, your treatment, etc.)

Feel free to elaborate on your experience: [text box]

Has anyone in your family or close to you experienced something significant with their health where they were required to seek care at a clinic or hospital?

If yes, currently or yes, but not recently:

Did they have any treatments?

Were they prescribed any medication?

How frequently did they go to the doctor during that time?

Overall, how was their experience with healthcare during that time? (This could mean their experience with their doctor, the clinic or hospital, their treatment, etc.)

Feel free to elaborate on their experience:

Has anyone else in your family or close to you experienced something significant with their health where they were required to seek care at a clinic or hospital?

[If yes, previous questions to repeat here]

**Section 2: Clinical Trials Participation**

One treatment option that people don’t typically recognize or think of as a treatment is enrollment in a clinical trial. This is especially true for new diseases like COVID-19 or rare diseases. Are you familiar with what a clinical trial is?
At any point in time, have you or anyone close to you participated in a clinical trial? Check all that apply.
If yes, what made you/someone close to you decide to participate? Check all that apply.

If yes, to the best of your ability, please answer the following questions about when you/someone close to you participated in a clinical trial.

What was the condition/diagnosis?

Approximately when did the clinical trial occur? ________________________________ (options: within the past 3 months, within the past year, within the past 1-3 years, more than 3 years ago)

How long did the clinical trial last? __________________________ (example: 6 months, 3 years, etc.)

If yes, How did your/the other person’s involvement in the clinical trial end? Check all that apply.

*[If no, skip to q below]*

Given the brief overview of a clinical trial above, if you had a condition that would allow you to join a clinical trial, would you be willing to participate?

If no, What are some reasons you might want to participate? Check all that apply.

If no, What are some reasons you might not want to participate? Check all that apply.

**Section 3: COVID-19 Clinical Trials Participation**

Typically, clinical trials only include people who are already sick. With COVID-19, clinical trials were preventive and included healthy people. Does this change whether you’d participate in a clinical trial?

Did you try to enroll in a clinical trial for COVID-19?

Would you encourage a family member or someone close to you to enroll in a clinical trial for COVID-19?

Have you experienced COVID-19?

If yes, did you receive a diagnosis of COVID-19?

Has anyone close to you experienced COVID-19?

If yes, did they receive a diagnosis of COVID-19?

**Section 4: Barriers**

What are some barriers you think people of color experience when participating in clinical trials? If you have participated in a clinical trial, use your own experience.

**Section 5: Solutions**

Given the barriers listed in the previous section, what can health systems do to reduce these barriers?

The following is a list of solutions people have suggested. Which do you think are good ideas?

[Card Sort] We want to wrap up by talking to you about potential solutions for improving clinical trial participation among people of color. The following are potential solutions that have been identified by others, and we would like you to sort them into these three categories:
1 – This solution would not make me want to participate
2 – This solution might make me want to participate
3 – This solution would definitely make me want to participate

You should sort these cards based only on your experience and/or your opinions. There is no right or wrong answer.

**Section 6: Demographics**

What is your age?

Are you of Hispanic, Latino, or Spanish origin?

What race do you identify as?

What gender do you identify as?

What type of insurance do you have?

What is your zip code?

**Appendix B: Response Codes**

| **Trial Staff Barrier** |
| --- |
| Anxiety about current diagnosis |
| Anxiety about participating |
| Believes current treatment is sufficient |
| General hesitance of research |
| Insurance issues |
| Lack of information/awareness |
| Lack of provider support |
| Lack of trust in care team |
| Language/communication issues |
| Logistics |
| Medical comorbidities |
| Not willing to go through additional procedures/treatment |
| Technological challenges |
| **Trial Staff Solution** |
| Change physician compensation model |
| Educate patients on disease basics and expected outcomes |
| Educate patients on research opportunities |
| Educate staff on implicit bias |
| Educate staff on the difference between health equity and health equality |
| Find ways to foster trust between the system and the communities served |
| Improve system-wide access to interpreter and/or translation services |
| Include more diverse populations in AAH marketing materials |
| Introduce incentives for staff who recruit patients |
| Invest in staffing sites with diverse patient populations |
| More transparency regarding trials that are being considered |
| Open different types of trials in which community members and patients are interested in participating |
| Provide staff with results from a cost-benefit analysis to understand importance of trial enrollment |
| Raise awareness of open trials through flyers, posters, etc. |
| Use patient success stories in recruitment |
| **Community Member Barrier** |
| Anxiety about current diagnosis |
| Anxiety about participating |
| Believes current treatment is sufficient |
| General hesitance of research |
| Insurance issues |
| Lack of financial incentive |
| Lack of information/awareness |
| Lack of trust in care team |
| Language barriers |
| Logistics |
| Not eligible due to medical conditions |
| Not willing to go through additional procedures/treatments |
| **Community Member Solution** |
| Improve access to interpreter and/or translation services within healthcare system |
| Improve diversity of clinical site staff in the health system |
| Improve trust between the healthcare system and the communities served |
| Include more diverse populations in marketing materials |
| Increase awareness of trial opportunities |
| Increase the healthcare system's presence in the community |
| Provide more education on diseases and treatments |
| Provide more time for staff to educate patients on clinical trials |
